# Cerebral Venous Thrombosis in a Patient With Smith-Magenis Syndrome

**DOI:** 10.7759/cureus.36858

**Published:** 2023-03-29

**Authors:** Hovra Zahoor, Ameer Hamza, Eboselumen Aigbe, Daniel Vather-Wu, Nilmarie Guzman

**Affiliations:** 1 Internal Medicine, HCA Florida Orange Park Hospital, Orange Park, USA

**Keywords:** cerebral venous sinus thrombosis (cvst), genetic metabolic disorders, hypercoagulation, cerebral venous and dural sinus thrombosis, smith magenis syndrome

## Abstract

Smith-Magenis syndrome (SMS) is a complex genetic developmental disorder characterized by distinctive physical features, cognitive impairment, developmental delay, and behavioral abnormalities. It is caused by a microdeletion of chromosome band 17p11.2 encoding for multiple genes including the Retinoic-acid-induced (RAI1) gene. RAI1 gene is expressed in many tissues, acting as a transcriptional regulator. It is a dosage-sensitive gene. The variants of the RAI1 gene have been explored with some contributing to systemic manifestations. The hematological manifestations such as venous thrombosis (VT) including cerebral venous thrombosis (CVT) have not been reported to date. We report a case of a 25-year-old female with SMS who presented with lethargy and gastrointestinal symptoms and was diagnosed with CVT. Our case highlights the risk of VT in patients with SMS and therefore holding a high index of suspicion for early diagnosis and management.

## Introduction

Smith-Magenis syndrome (SMS) is a complex genetic developmental disorder. It is typified by a microdeletion of chromosome band 17p11.2 which encodes for multiple genes including the retinoic-acid-induced 1 (RAI1) gene. SMS is characterized by distinctive physical features (particularly coarse facial features that progress with age), cognitive impairment, behavioral abnormalities, developmental delay, sleep disturbance, and childhood-onset abdominal obesity. The majority of individuals function in the mild-to-moderate range of intellectual disability [1]. SMS is a rare disorder with continuous exploration of multi-systemic manifestations. Multisystemic manifestations including cardiovascular (congenital heart defects), genitourinary malformations, ocular (microcornea, strabismus), otolaryngological (sensorineural hearing loss), and malignancy are known [1]. However, hematological manifestations such as venous thrombosis (VT) including cerebral venous thrombosis (CVT) have not been reported to date. Here, we report a case of a 25-year-old female with SMS who was diagnosed with CVT in absence of any classic risk factors for VT and any acquired or inherited hypercoagulable disorders. Our aim is to contribute to the literature in regard to the multi-systemic manifestations of SMS.

## Case presentation

A 25-year-old female with a medical history significant for SMS and chronic anemia was brought to the emergency department (ED) by her mother for complaints of lethargy, vomiting, diarrhea, and abdominal pain for the past few days. On detailed interviewing, the patient's mother reported that the patient has had nausea and vomiting for the past four days. This was associated with low-grade fever, abdominal discomfort, and 1-2 episodes of non-bloody diarrhea. The patient's mother reported that the patient was diagnosed with SMS in early childhood, causing the severe developmental delay. She reported patient's mental age was 12. She stated she wasn't initially concerned about the patient's symptoms as she attributed it to some viral illness. However, per the mother, the patient wasn't acting her usual self and was more lethargic which prompted her to bring the patient to the ED.

In the ED, the patient's Glasgow Coma Scale (GCS) [2] was 13. Vital signs were normal. A physical exam revealed mild abdominal tenderness and lethargy, otherwise unremarkable. No evidence of weakness, numbness, or cranial nerve abnormalities.

**Figure 1:**
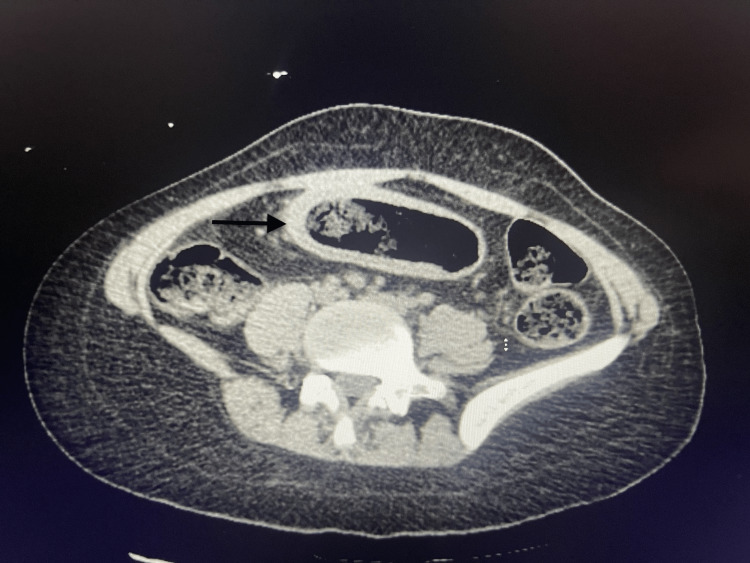
CT abdomen/pelvis without contrast showed stool throughout the colon with circumferential thickening (arrow) of the wall of the rectum and sigmoid colon likely proctocolitis. CT: computed tomography

Initial work-up revealed hemoglobin of 5.9 but normal white blood cell count and platelet count. The comprehensive metabolic panel was within normal limits. COVID (Coronavirus-19 disease) test was negative. Computed tomography (CT) abdomen pelvis without contrast revealed signs of proctocolitis (Figure 1). The patient received intravenous (IV) fluids, two units of packed red blood cells, and IV antibiotics - ceftriaxone and metronidazole.

Further work-up for microcytic anemia was ordered which included an iron panel. Gastroenterology was consulted given her microcytic anemia and the CT findings. She underwent upper endoscopy as well as a colonoscopy which showed no evidence of bleeding. However, reflux esophagitis and congested mucosa on the sigmoid colon were noted. The patient has initiated on Protonix 40 mg orally daily and IV antibiotics were continued. The iron panel revealed evidence of iron deficiency and the patient was thereby started on iron supplementation.

**Figure 2:**
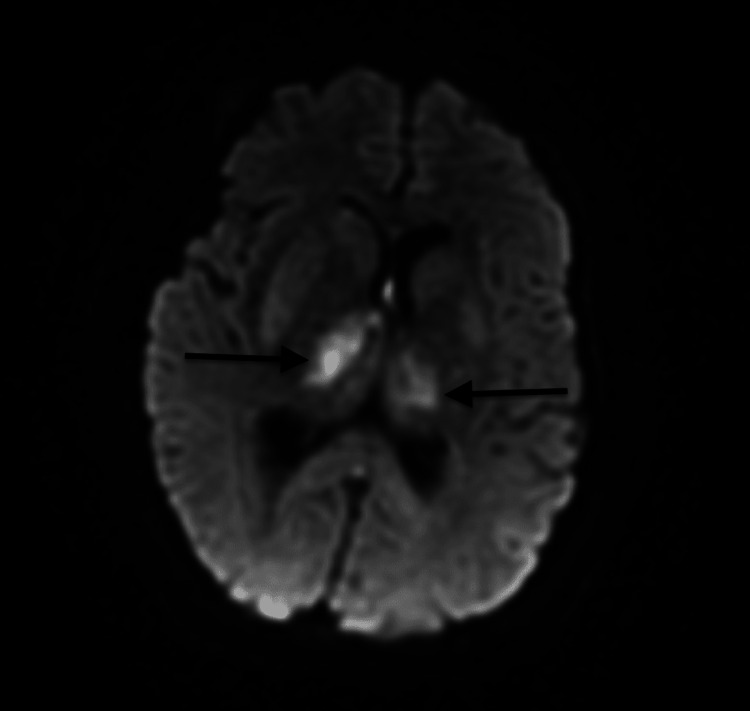
MRI brain with/without contrast revealed secondary venous infarcts of bilateral thalami and right basal ganglia. MRI: magnetic resonance imaging

On hospital day 2, the patient was noted to be increasingly altered. A stroke alert was initiated. No tenecteplase was recommended given the unclear last known well. CT brain without contrast revealed findings concerning CVT and venous infarct. CT brain venogram revealed occlusion of the inferior sagittal sinus and bilateral deep cerebral veins. Magnetic resonance imaging (MRI) brain with/without contrast (Figure 2) revealed venous infarcts of bilateral thalami and right basal ganglia in addition to CVT. Neuro-interventionalist recommended anticoagulation with IV heparin and no surgical intervention due to patency of the torcula and right transverse/sigmoid system.

Hematology was consulted. Lupus anticoagulant, antiphospholipid, antinuclear, and antineutrophil cytoplasmic antibodies were negative. Fibrinogen and D-dimer were within normal limits. The patient’s GCS worsened (8) on day 4; she was intubated for airway protection. Electroencephalogram (EEG) revealed interictal discharges, thereby, levetiracetam was initiated. The patient had a gradual improvement in neurological status and was extubated on day 10. The patient was transitioned to apixaban prior to discharge to a rehabilitation facility. Extensive work-up outpatient was negative for any inherited or acquired hypercoagulable disorders. The patient was discharged from the rehabilitation facility with remarkable improvement.

## Discussion

SMS is a rare genetic developmental disorder with a birth incidence between 1:15,000 and 1:25,000 [1]. It is characterized by distinctive physical features, cognitive impairment, developmental delay, and behavioral abnormalities. It is caused by 17p11.2 interstitial deletions (90%), an encompassing multitude of genes including RAI1, or by pathogenic variants in RAI1 itself (10%) [1]. RAI1 is a dosage-sensitive gene. It is expressed in many tissues, acting as a transcriptional regulator. The diagnosis of SMS is established in a patient who has suggestive clinical features and either a heterozygous deletion at chromosome 17p11.2 that includes RAI1 or a heterozygous intragenic RAI1 pathogenic variant. The variants of the RAI1 gene have been explored with some contributing to systemic manifestation. Multisystemic manifestations that have been reported include congenital heart defects (25-45% of patients), genitourinary malformations (14-35% of patients), sensorineural hearing loss, ocular manifestations (microcornea, strabismus), musculoskeletal (scoliosis), and malignancy [1]. The hematological manifestations such as VT including CVT have not been reported to date. It is important to be aware of the association of VT with SMS as VT especially CVT and pulmonary embolism are life-threatening. Reporting this association will create awareness among physicians to hold a high index of suspicion of these in patients with SMS and thereby help in early diagnosis and management.

CVT is a thrombosis of cerebral veins. The incidence of CVT is 0.22-1.32/100,000/year [3] accounting for 0.5% of all strokes [4]. Clinical presentation of CVT, however, is variable and includes headache (present in 90% of cases), stroke-like symptoms (40% of patients present acutely within 48 hours), craniofacial pain, and seizures [5]. Diagnosis requires a high index of clinical suspicion in conjunction with neuro-radiological diagnostic support (brain MRI and MR venography or cranial CT with CT venography). Risk factors for CVT include genetic hypercoagulable disorders, malignancy, pregnancy, puerperium, estrogen-containing medications, and autoimmune disorders [6]. Up to 90% of patients with CVT have at least one risk factor for venous thromboembolism (VTE), and 30% of the patient have thrombophilias (genetic or acquired) [7]. Female-specific risk factors (i.e. estrogen-containing contraceptives, puerperium, and pregnancy) are more important in younger age groups and malignancy is more common in older age groups [6].

Our patient did not exhibit classic risk factors for VT, had no history of prior VT or family history of hypercoagulable disorders, and was not on any estrogen-containing medications. Workup was negative for any inherited or acquired hypercoagulable disorders and autoimmune conditions including vasculitis. Our case suggests the possible role of gene variants in SMS contributing to VT. Further research in this direction is required as it will guide the management approach and duration of anticoagulation.

## Conclusions

Literature regarding multisystemic manifestations of SMS is limited. Our case suggests the association of hematological manifestations such as CVT with SMS and the possible role of RAI1 gene variants in SMS contributing to VT. Further research is required to determine pathogenic variants of RAI1 contributing to VT. Our case also highlights the importance of holding a high index of suspicion for CVT in patients with SMS, especially those who present with altered mental status or stroke-like symptoms. This should prompt the immediate ordering of neurodiagnostic tests as early diagnosis and management with the initiation of anticoagulation or mechanical thrombectomy is essential for better outcomes.
